# Effect of *Jatropha curcas* Peptide Fractions on the Angiotensin I-Converting Enzyme Inhibitory Activity

**DOI:** 10.1155/2013/541947

**Published:** 2013-10-10

**Authors:** Maira R. Segura-Campos, Fanny Peralta-González, Arturo Castellanos-Ruelas, Luis A. Chel-Guerrero, David A. Betancur-Ancona

**Affiliations:** Facultad de Ingeniería Química, Universidad Autónoma de Yucatán, Periférico Nte. Km. 33.5, Tablaje Catastral 13615, Col. Chuburná de Hidalgo Inn, 97203 Mérida, YUC, Mexico

## Abstract

Hypertension is one of the most common worldwide diseases in humans. Angiotensin I-converting enzyme (ACE) plays an important role in regulating blood pressure and hypertension. An evaluation was done on the effect of Alcalase hydrolysis of defatted *Jatropha curcas* kernel meal on ACE inhibitory activity in the resulting hydrolysate and its purified fractions. Alcalase exhibited broad specificity and produced a protein hydrolysate with a 21.35% degree of hydrolysis and 34.87% ACE inhibition. Ultrafiltration of the hydrolysate produced peptide fractions with increased biological activity (24.46–61.41%). Hydrophobic residues contributed substantially to the peptides' inhibitory potency. The 5–10 and <1 kDa fractions were selected for further fractionation by gel filtration chromatography. ACE inhibitory activity (%) ranged from 22.66 to 45.96% with the 5–10 kDa ultrafiltered fraction and from 36.91 to 55.83% with the <1 kDa ultrafiltered fraction. The highest ACE inhibitory activity was observed in *F*2 (IC_50_ = 6.7 **μ**g/mL) from the 5–10 kDa fraction and *F*1 (IC_50_ = 4.78 **μ**g/mL) from the <1 kDa fraction. ACE inhibitory fractions from *Jatropha* kernel have potential applications in alternative hypertension therapies, adding a new application for the *Jatropha* plant protein fraction and improving the financial viability and sustainability of a Jatropha-based biodiesel industry.

## 1. Introduction

Jatropha is a genus of approximately 175 succulent plants, shrubs, and trees from the family Euphorbiaceae. Jatropha plant has been used traditionally for medical purposes, for both humans and animals. The species that is most widely studied in a nutritional context is *Jatropha curcas*. The seed contains kernel and shell with an average ratio of 62.2 : 37.7. The kernel has higher crude protein (22–28%) and oil contents (54–58%) compared to the shell (4–6% and 0.8–1.4%, resp.) [1]. *J. curcas* seeds contain about 300–350 g kg^−1^ oil, which can be used directly as fuel or, in its transesterified form, as a substitute for diesel. Large-scale planting of Jatropha has already taken place or is currently being planned in India, China, Madagascar, Myanmar, and many other developing countries, aiming to use the oil as biodiesel [2]. 

Toxic and nontoxic genotypes of *J. curcas* are available. The toxic genotype is prevalent throughout the world, and the nontoxic genotype exists only in Mexico. The use in animal nutrition of defatted Jatropha meals and protein isolate prepared from the toxic genotype is restricted due to the presence of antinutritional and toxic factors (phorbol esters). The phorbol esters are reported to mimic the action of diacyl-glycerol, activator of protein kinase C, which regulates different signal transduction pathways. Interference with the activity of protein kinase C affects a number of processes including phospholipid and protein synthesis, enzyme activities, DNA synthesis, phosphorylation of proteins, cell differentiation, and gene expression. They are also cocarcinogens and have purgative and skin-irritant activities. In humans, accidental poisoning by Jatropha seeds has been reported to elicit giddiness, vomiting, and diarrhea, and mortality has also been reported in a number of animal species. Similar to the toxic genotype, the nontoxic *J. curcas* kernels are rich in oil (55–58%) and protein (26–29%). The essential amino acids composition of defatted Jatropha meal, except lysine, is similar to the toxic genotype, and levels of other essential amino acids are similar to the FAO reference protein pattern. Defatted Jatropha meal from the nontoxic genotype is free of phorbol esters, but it contains trypsin inhibitor, lectin, and phytate at the same levels as the meal from the toxic genotype [3].

In recent years, the global industrialization and the increased demand for livestock products needed to meet human food requirements have greatly increased the pressure on agricultural land and on the environment. Since the production of animal protein does not meet the requirement of the market, the search for new protein sources that do not compete with the ones that are now available is of the upmost importance. Nonedible oil seeds have the highest potential and are the preferred choice as protein and other nutrients for livestock, provided that they are free of toxic and antinutritional factors. A promising oilseed plant is *Jatropha curcas* that has advantages over other oilseed plants (e.g., *Pongamia pinnata, Simarouba glauca, Ricinus communis, *and* Azadirachta indica*), since it is adaptable to a wide range of agroclimatic conditions [4]. Large-scale agronomical production units of Jatropha have being initiated in the past 5–10 years with a projected worldwide cultivation of 12.8 million hectares yielding 2 t/ha of oil by 2015 (Global Exchange for Social Investment market study). In the future, this will provide high amounts of pressed seed cake or kernel meal as byproducts, which are rich in high-quality protein, as mentioned above. These byproducts can be used in animal nutrition after detoxification and could also be a source for various bioactive protein molecules having a wide range of activities [5].

During biodiesel production, the Jatropha seeds are crushed in a mechanical press to obtain oil and a seed cake containing 7–10% residual oil and 22–24% crude protein. The seed cake could be sieved to remove shells, and the sieved material could be extracted with hexane or petroleum benzene to recover oil and to obtain the remaining defatted Jatropha kernel meal (JKM) [6]. Production of *J. curcas* protein isolate from JKM has been described as a way to reduce the antinutritional and toxic components. Since *J. curcas* protein isolate is a good substrate for the production of protein hydrolysates as a source of bioactive peptides with beneficial biological activities, the objective of the present study was to hydrolyze *Jatropha curcas* protein isolate with Alcalase in order to identify and quantify ACE-inhibitory activity in the protein hydrolysates obtained.

## 2. Materials and Methods

### 2.1. Materials

*Jatropha curcas* L. seeds were obtained from Centro de Investigación en Biotecnología Aplicada, IPN in Tepetitla, Tlaxcala, México. Reagents were of analytical grade and purchased from J. T. Baker (Phillipsburg, NJ, USA), Sigma (Sigma Chemical Co., St. Louis, MO, USA), Merck (Darmstadt, Germany), and Bio-Rad (Bio-Rad Laboratories, Inc. Hercules, CA, USA). Angiotensin converting enzyme from rabbit lung (2 units/mg protein) was purchased from Sigma (A6778 Sigma Chemical Co., St. Louis, MO, USA). The Alcalase 2.4L enzyme was purchased from Novo Laboratories (Copenhagen, Denmark). Alcalase 2.4L is an endopeptidase from *Bacillus licheniformis*, with subtilisin Carlsberg as the major enzyme component and a specific activity of 2.4 Anson units (AU) per milliliter. 

### 2.2. Defatted Jatropha Kernel Meal

Seed cake was produced from 6 kg *J. curcas* seed by first removing all impurities and damaged seeds and then crushing them in a mechanical press (Azteca model, Mérida, México). The seed cake was sieved to remove shells, and the sieved material was extracted with hexane in a Soxhlet system for 8 h (AOAC [7], method 920.39) to recover oil and to obtain the remaining Jatropha kernel meal (JKM). The JKM, obtained after solvent extraction of kernels (free of shells), was milled in a Cyclotec 1093 (Tecator, Sweden) mill. Particle size was obtained forcing the JKM through an 80 and 100-mesh screen. 

### 2.3. *Jatropha curcas* Protein Isolate

The JKM was processed using the wet fractionation method of Betancur-Ancona et al. [8]. Briefly, JKM was suspended in distilled water at a 1 : 6 (w/v) ratio, pH was adjusted to 10.5 with 1 M NaOH, and the dispersion was stirred for 1 h at 400 rpm with a mechanical agitator (Caframo Rz-1, Heidolph Schwabach, Germany). This suspension was wet milled with a Kitchen-Aid mill, and the fiber solids were separated from the starch and protein mix by straining through 80- and 150-mesh sieves and washing the residue five times with distilled water. The protein-starch suspension was allowed to stand for 30 min at room temperature to starch sedimentation and after protein fraction was decanted to recover the solubilized protein. The pH of the separated solubilized proteins was adjusted to their isoelectric point (5.5) with 1 M HCl. The suspension was then centrifuged at 1317 ×g for 12 min (Mistral 3000i, Curtin Matheson Sci.), the supernatants were discarded, and the precipitates were freeze dried at −47°C and 13 × 10^−3^ mbar. Until use, the protein isolate was stored in plastic containers at room temperature.

### 2.4. Enzymatic Hydrolysis

*J. curcas* protein isolate prepared as previously described was hydrolyzed using Alcalase 2.4L FG according to Yust et al. [9] method. The response variable was the degree of hydrolysis (DH). Hydrolysis was done under controlled conditions (temperature, pH, and stirring) in a 1000 mL reaction vessel equipped with a stirrer, thermometer, and a pH electrode. Isolates were suspended in distilled water to produce a 4% (w/v) protein solution. This solution was equilibrated at optimum temperature and pH before adding the respective enzyme. Protease was then added to the solution at a ratio 1 : 10 enzyme/substrate. Hydrolysis conditions were 60 min of hydrolysis time, 50°C of temperature, pH 8.0, and a specific activity of 0.3 AU per milliliter from Alcalase. The pH was kept constant by adding 1.0 M NaOH or 1.0 M HCl during hydrolysis. The reaction was stopped heating at 80°C for 20 min, followed by centrifuging at 9880 ×g for 20 min to remove the insoluble portion. Hydrolysates protein content was determined according to Lowry et al. [10].

### 2.5. Degree of Hydrolysis

Degree of hydrolysis was calculated by determining free amino groups with o-phthaldialdehyde following the equation described by Nielsen et al. [11]. Consider

(1) $$\begin{array}{c} DH=\frac{h}{h_{\text{tot}}}*100, \end{array}$$

where  *h*_tot_ is the total number of peptide bonds per protein equivalent and *h* is the number of hydrolyzed bonds. 

The *h*_tot_ factor is dependent on the raw material amino acid composition, and it was determined by reverse-phase high performance liquid chromatography (RP-HPLC) according to Alaiz et al. [12]. Samples (2–4 mg protein) were treated with 4 mL of 6 mol equi L^−1^ HCl, placed in hydrolysis tubes and gassed with nitrogen at 110°C for 24 h. They were then dried in a rotavapor and suspended in 1 mol L^−1^ sodium borate buffer at pH 9.0. Amino acid derivatization was performed at 50°C using diethyl ethoxymethylenemalonate. Amino acids were separated using HPLC with a reversed phase column (300 × 3.9 mm, Nova Pack C18, 4 mm; Waters) and a binary gradient system with 25 mmol L^−1^ sodium acetate containing (A) 0.02 g L^−1^ sodium azide at pH 6.0 and (B) acetonitrile as solvent. The flow rate was 0.9 mL min^−1^, and the elution gradient was: time 0.0–3.0 min, linear gradient A : B (91 : 9) to A-B (86 : 14); time 3.0–13.0 min, elution with A-B (86–14); time 13.0–30.0 min, linear gradient A-B (86 : 14) to A-B (69 : 31); time 30.0–35.0 min, elution with A-B (69 : 31). 

### 2.6. Hydrolysate Fractionation by Ultrafiltration

Alcalase hydrolysate was fractionated by ultrafiltration (UF) according to Cho et al. [13] using a high performance ultrafiltration cell (Model 2000, Millipore). Five fractions were prepared using four molecular weight cut-off (MWCO) membranes: 1 kDa, 3 kDa, 5 kDa, and 10 kDa. Soluble fractions prepared by centrifuging (9880 ×g for 20 min) were passed through the membrane starting with the largest MWCO membrane cartridge (10 kDa). The retentate and permeate were collected separately, and the retentate recirculate into the feed until maximum permeate yield was reached, as indicated by a decreased permeate flow rate. Permeate from the 10 kDa membrane was then filtered through the 5 kDa membrane with recirculation until maximum permeate yield was reached. The 5 kDa permeate was then processed with the 3 kDa membrane and the 3 kDa permeate with the 1 kDa membrane. This process minimized contamination of the larger molecular weight fractions with smaller molecular weight fractions while producing enough retentates and permeates for the following analyses. Five ultrafiltered peptide fractions were prepared and designated as >10 kDa (10 kDa retentate), 5–10 kDa (10 kDa permeate-5 kDa retentate), 3–5 kDa (5 kDa permeate-3 kDa retentate), 1–3 kDa (3 kDa permeate-1 kDa retentate), and <1 kDa (1 kDa permeate). 

### 2.7. ACE-I Inhibitory Activity

ACE-I inhibitory activity in the hydrolysate and its ultrafiltered peptide fractions were analyzed following Hayakari et al. [14] method, which is based on the fact that ACE-I hydrolyzes hippuryl-L-histidyl-L-leucine (HHL) yielding hippuric acid and L-histidyl-L-leucine. This method relies on the colorimetric reaction of hippuric acid with 2,4,6-trichloro-S-triazine (TT) in a 0.5 mL incubation mixture containing 40 *μ*mol potassium phosphate buffer (pH 8.3), 300 *μ*mol sodium chloride, 40 *μ*mol 3% HHL in potassium phosphate buffer (pH 8.3), and 100 mU/mL ACE-I. This mixture was incubated at 37°C/45 min and the reaction terminated by addition of TT (3% v/v) in dioxane and 3 mL 0.2 M potassium phosphate buffer (pH 8.3). After centrifuging the reaction mixture at 10,000 ×g for 10 min, enzymatic activity was determined in the supernatant by measuring absorbance at 382 nm. All determinations were done by triplicate. ACE-I inhibitory activity was quantified by a regression analysis of ACE-I inhibitory activity (%) versus peptide concentration. IC_50_ values (i.e., the peptide concentration in *μ*g protein/mL required to produce 50% ACE-I inhibition under the described conditions) were defined and calculated as follows:

(2) $$\begin{array}{c} \text{ACE-I}\;\text{inhibitory}\;\text{activity}\;\left(\%\right)=\frac{\left(A-B\right)}{\left(A-C\right)}\times 100, \end{array}$$

where *A* represents absorbance in the presence of ACE-I sample,  *B* represents absorbance of the control, and  *C* represents absorbance of the reaction blank.

(3) $$\begin{array}{c} {\text{IC}}_{50}=\frac{\left(50-b\right)}{m}, \end{array}$$

where *b* is the intersection and *m* is the slope. 

### 2.8. Amino Acid Composition

Amino acid composition was determined in the ultrafiltered fractions with the highest biological activity according to Alaiz et al. [12] method. Samples (2–4 mg protein) were treated with 4 mL of 6 mol equi L^−1^ HCl, placed in hydrolysis tubes and gassed with nitrogen at 110°C for 24 h. They were then dried in a rotavapor and suspended in 1 mol L^−1^ sodium borate buffer at pH 9.0. Amino acid derivatization was performed at 50°C using diethyl ethoxymethylenemalonate. Amino acids were separated using HPLC with a reversed phase column (300 × 3.9 mm, Nova Pack C18, 4 mm; Waters) and a binary gradient system with 25 mmol L^−1^ sodium acetate containing (A) 0.02 g L^−1^ sodium azide at pH 6.0 and (B) acetonitrile as solvent. The flow rate was 0.9 mL min^−1^, and the elution gradient was time 0.0–3.0 min, linear gradient A : B (91 : 9) to A-B (86 : 14); time 3.0–13.0 min, elution with A-B (86–14); time 13.0–30.0 min, linear gradient A-B (86 : 14) to A-B (69 : 31); and time 30.0–35.0 min, elution with A-B (69 : 31). Tryptophan content was determined according to Yust et al. [15] method.

### 2.9. G-50 Gel Filtration Chromatography

After filtration through 10, 5, 3, and 1 kDa membranes in a high performance ultrafiltration cell, 10 mL of the fraction with highest ACE-I inhibitory activity was injected into a Sephadex G-50 gel filtration column (3 cm × 79 cm) at a flow rate of 25 mL/h of 50 mM ammonium bicarbonate (pH 9.1). The resulting fractions were collected for to assay ACE-I inhibitory activity [14]. Peptide molecular masses were determined referring to a calibration curve running molecular mass markers on the Sephadex G-50 under identical conditions as those used for the test samples. Molecular mass standards were thyroglobulin (670 kDa), bovine gamma globulin (158 kDa), equine myoglobin (17 kDa), vitamin B_12_ (1.35 kDa), and Thr-Gln (0.25 kDa). Fractions selected for further purification of peptides were pooled and lyophilized before RP-HPLC. 

### 2.10. Statistical Analysis

All results were analyzed in triplicate using descriptive statistics estimating measures of center and of variation. Six lots of 1 Kg from *J. curcas* seeds were processed to obtain the defatted Jatropha kernel meal and the protein isolate. From the last one, a composite sample was obtained and the enzymatic hydrolysis was done by three replicates. Biological potential from Jatropha hydrolysate and their purified fractions were all done by triplicate too. One-way ANOVAs were performed to evaluate *in vitro* ACE-I inhibitory activity. A Duncan multiple range test was used to determine differences between treatments. All analyses were performed according to Montgomery [16] and processed using the Statgraphics Plus version 5.1 software.

## 3. Results and Discussion

### 3.1. *Jatropha curcas* Protein Isolate

Protein isolates, as the name suggests, are the concentrated forms of plant proteins, generally prepared by solubilizing proteins and removing nonprotein ingredients. According to Devappa et al. [6], the nitrogen solubility profile of JKM, as a function of pH, has a U shape, which is typical for oil seed proteins. Jatropha proteins have a minimum solubility in water at pH 5.5 and increased solubility at acidic and alkaline pH values, with maximum nitrogen solubility at pH 10.5. According to these principles and using Betancur-Ancona et al. [8] method, a *J. curcas* protein isolate was obtained. The isolate had a protein content of 92.23%. Its protein content was similar to that reported by Gallegos-Tintoré et al. [17] who used *J. curcas* protein isolates (87% of protein content) as substrate to prepare Alcalase protein hydrolysates with antioxidant activity. In this sense, Makkar et al. [18] reported the preparation of protein isolate from Jatropha screw-pressed cake (JC) prepared by pressing whole seeds and containing a high level of shells (approximately 50%). The residual oil content was 7%. They also prepared protein isolate from defatted Jatropha screw-pressed cake (DJC) containing <0.5% fat. The recovery of protein was the highest (29.5 and 33%) when proteins were extracted at pH 11 (60°C) and precipitated at pH 4 in cold for JC and DJC, respectively. The protein contents of the protein isolates were 76.0 and 87.0% for JC and DJC, respectively, which were lower to the results in the present study. 

Devappa et al. [6] reported the preparation of protein isolate from (a) Jatropha meal (JM, fat 0.8% oil) obtained after passing hexane-defatted Jatropha seed cake (obtained after mechanical pressing), through a mesh to remove the majority of hulls, and (b) JKM (0.8% oil). In brief, both JM and JKM were extracted at alkaline pH (10.5) by injecting steam (92 ± 2°C) for 10 min. The proteins in the cooled supernatant were precipitated at pH 5.5 to get wet protein isolate, which was neutralized and spray dried to obtain Jatropha protein isolates. These isolates had protein contents of 96-97%, which were similar to the results previously described in this paper. 

### 3.2. Enzymatic Hydrolysis of  *J. curcas* Protein Isolate

Alcalase was used to produce a *J. curcas* protein hydrolysate. The bacterial endoprotease Alcalase is limited by its specificity, resulting in DHs no higher than 20 to 25%, depending on the substrate, but it can attain these DHs in a relatively short time under moderate conditions. In the present study, Alcalase exhibited broad specificity and produced a protein hydrolysate with DH value from 21.35%. The *J. curcas* protein hydrolysate had a lower DH value than that reported for rapeseed protein hydrolysates (60% DH) produced with a mixture of Alcalase and flavourzyme during 180 min [19] but higher than the reported values for mung bean protein hydrolysates produced with Alcalase (10% DH) for 10 h [20]. The variation in DH is probably the result of protease specificity and of protein source since Alcalase is an industrial alkaline protease produced from *Bacillus licheniformis*, the main enzyme component of which (serine endopeptidase subtilisin, Carlsberg) presents broad specificity and hydrolyzes most peptide bonds, with a preference for those containing aromatic amino acid residues [21]. ACE-I prefers substrates or competitive inhibitors containing hydrophobic amino acid (aromatic or branched lateral chain) residues [20]. Alcalase is therefore very suitable for production of bioactive peptides, such as those with ACE-I inhibitory activity. According to Pedroche et al. [22], the controlled liberation of biologically active peptides from protein by enzymatic hydrolysis is one of the most promising trends concerning medical applications of the protein hydrolysates with degree of hydrolysis higher than 10% while hydrolysates with a low degree of hydrolysis (lower than 10%) are used for the improvement of functional properties of flours or protein isolates. Therefore, the results suggest (DH = 21.35%) that *J. curcas* protein is an appropriate substrate for producing these bioactive peptides when hydrolyzed with Alcalase.

### 3.3. ACE-I Inhibitory Activity

ACE-I inhibitory activity of the *J. curcas* protein hydrolysates was measured and calculated as ACE-I inhibition percentage. The protein hydrolysate obtained from *J. curcas* protein isolate showed an ACE-I inhibition percentage of 34.87% at protein concentration of 0.02 mg/mL. The ACE-I inhibitory activity observed here was lower than that reported for yak milk casein hydrolyzed with Alcalase for 240 min (79.5%) [23] but similar to the one obtained for protein hydrolysates from albumin 1 and globulin of amaranth (*Amaranthus hypochondriacus*) (5–50%) by Tovar-Pérez et al. [24]. 

**Figure 1:**
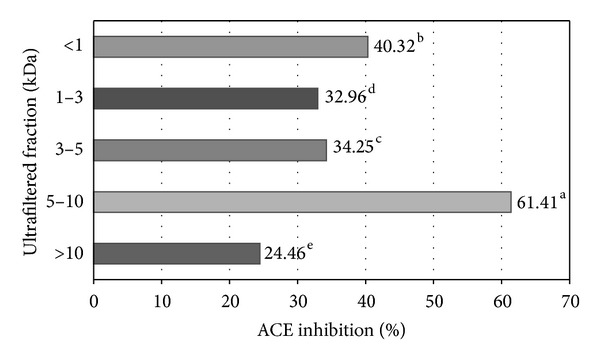
IC_50_ values of peptide fractions obtained by ultrafiltration from *J. curcas* protein hydrolysates. ^a–e^Different superscripts letters indicate statistical difference (*P* < 0.05). Mean of three replicates.

Matsufuji et al. [25] concluded that peptides produced by enzymes such as Alcalase, which exhibit ACE-I inhibitory activity, may resist digestion by gastrointestinal proteases and therefore be absorbed in the small intestine, a quality also observed in a number of spontaneously hypertensive rats (SHRs) studies. Based on the above, it is probable that the *J. curcas* protein hydrolysates produced here with Alcalase, exhibiting ACE-I inhibitory activity, are capable of resisting gastrointestinal proteases and are therefore appropriate for their application in food systems (e.g., functional foods) focused on people suffering arterial hypertension disorders. Ultrafiltration of the Alcalase hydrolysate produced peptide fractions with increased biological activity. ACE-I inhibition percentage of peptide fractions ranged from 24.46 to 61.41% (Figure 1). ACE-I inhibition percentage was different (*P* < 0.05) depending on peptide fraction molecular weight, with the lowest activity being in the >10 kDa fractions and the highest in the 5–10 kDa fractions. Results also confirm the fact that ultrafiltration membranes can be used to enrich specific peptide fractions. Je et al. [26] informed that the fraction having a molecular mass below 1 kDa from Alaska Pollack (*Theragra chalcogramma*) frame protein hydrolysate showed the highest ACE-inhibitory activity (87.62%, IC_50_ = 457 *μ*g/mL) than that *F*1–3 kDa (78.95%), *F*3–5 kDa (73.84%), *F*5–10 kDa (68.24%), and *F*10–30 kDa (59.60%) fractions. These results suggest that ultrafiltration is a promising way of enriching ACE-inhibitory peptides derived from *J. curcas* proteins. This has been demonstrated with other raw materials. For instance, ultrafiltered thermolysin digest from dried bonito had more potent antihypertensive effects in hypertensive subjects than the source hydrolysate [27]. Mao et al. [28] concluded that the lower was the molecular weight of the yak casein hydrolysate fraction, the higher was its ACE-I inhibitory activity, and the molecular weight of the most effective fraction was below 6 kDa (85.4%).

### 3.4. Amino Acid Composition

Fractions with the highest ACE-I inhibitory activity purified by ultrafiltration were analyzed to produce an amino acid profile. During hydrolysis, asparagine and glutamine fully converted to aspartic acid and glutamic acid, respectively; the data for asparagine and/or aspartic acid were therefore reported as Asx while those for glutamine and/or glutamic acid were reported as Glx. The higher ACE-I inhibitory activity exhibited by the 5–10 kDa fraction (61.41%) compared to the <1 kDa fraction (40.32%) was probably due to its higher concentration of hydrophobic (39.11 g/100 g) and neutral (26.40 g/100 g) amino acids (Table 1).

**Figure 2:**
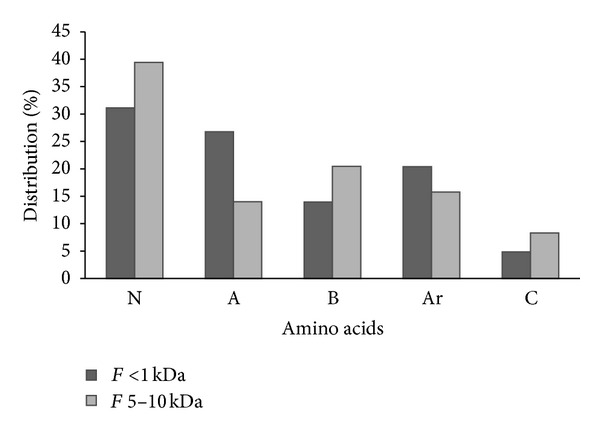
Amino acid distribution in the <1 kDa and 5–10 kDa ultrafiltered fraction from *J. curcas* protein hydrolysates. N: neutral amino acids (including Gly, Ala, Ser, Thr, Val, Leu, and Ile). A: acid amino acids (including Asp and Glu). B: basic amino acids (including Lys, His, and Arg). Ar: aromatic amino acids (including Phe, Tyr, and Trp). C: cyclic amino acids (including Pro).

Comparison of amino acid composition and the biological potential between the 5–10 kDa and <1 kDa fractions showed the most active fraction to be the 5–10 kDa, which had abundant neutral (e.g., Ser, Gly, Thr, Ala, and Ile), basics (e.g., His and Arg), and cyclic amino acids (e.g., Pro) (Figure 2). According to Wu et al. [29], amino acids such as Pro are preferred in the carboxyl terminus of active tripeptides for their low lipophilicity and high steric and electronic properties. Similar observations have been made for an orientase hydrolysate with considerable ACE-I inhibition attributed to its high basic and aromatic amino acids contents [30]. Based on the above, the higher ACE-I inhibitory potential of the 5–10 kDa fraction from the *J. curcas* hydrolysate can be attributed to the steric properties of its aromatic amino acids and the lipophilicity and electronic properties of its cyclic amino acids. 

Of the neutral amino acids, Tyr was higher in 5–10 kDa (8.17 g/100 g) compared to <1 kDa fraction (7.87 g/100 g). This supports the importance of aromatic residues in a peptides' biological potential, probably due to their high steric properties and low lipophilicity. The Tyr amino acid has been reported in peptides from milk (e.g., Tyr-Pro-Tyr-Tyr) isolated by a combination of lactic acid bacteria fermentation and flavourzyme hydrolysis. These peptides are bioavailable and exhibit *in vitro* (90.9 *μ*M) and *in vivo* ACE-I inhibitory activity, the latter in the form of reduced hypertension in SHR (15.9 mm Hg reduction in SBP) [31]. The 5–10 kDa fraction also had a higher content in Met (0.84 g/100 g), Ile (8.73 g/100 g), Ala (7.60 g/100 g), and Lys (3.78 g/100 g) all of which could have significantly increased its relative ACE-I inhibition activity. The high hydrophobic amino acid (particularly aromatic side chains) content in the 5–10 kDa fraction may therefore make a substantial contribution to its fractions' ACE inhibitory activity by blocking angiotensin II production.

### 3.5. Gel Filtration Chromatography of Ultrafiltered Peptide Fractions from *J. curcas* Protein Hydrolysate

**Figure 3:**
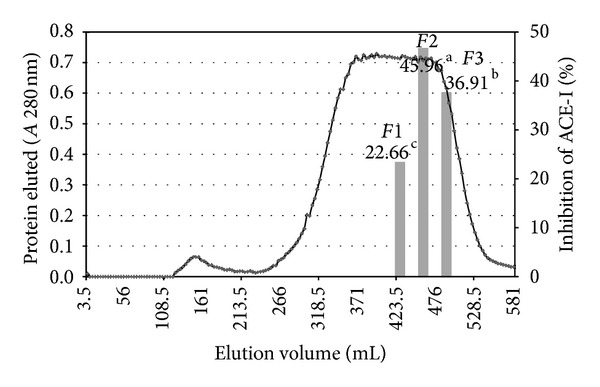
Elution profile of the 5–10 kDa ultrafiltration fraction of the *J. curcas* protein hydrolysate purified in a Sephadex G-50 gel filtration column. ^a–e^Different superscripts letters indicate statistical difference (*P* < 0.05). Mean of three replicates.

**Figure 4:**
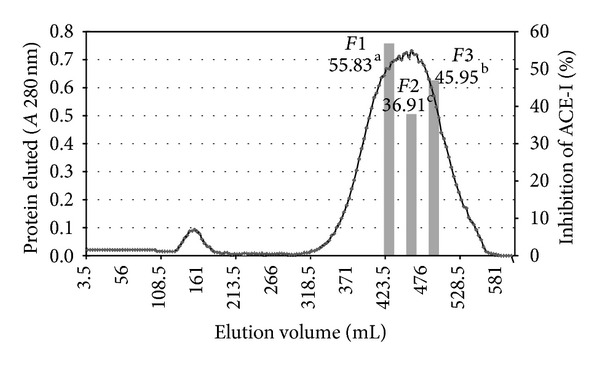
Elution profile of the <1 kDa ultrafiltration fraction of the *J. curcas* protein hydrolysate purified in a Sephadex G-50 gel filtration column. ^a–e^Different superscripts letters indicate statistical difference (*P* < 0.05). Mean of three replicates.

Since they exhibited the highest ACE-I inhibitory activity, the 5–10 and <1 kDa fraction were selected for further fractionation. Gel filtration chromatography (Sephadex G-50 column) was used to generate a molecular weight profile of the 5–10 and <1 kDa fraction. The profile was typical of a protein hydrolysate formed by a pool of peptides, with gradually decreasing molecular masses. Elution volumes between 406 and 518 mL included free amino acids and peptides with molecular masses ranging from 3.6 to 0.4 kDa. This range was fractionated into three fractions and ACE-I inhibitory activity determined for each. Fractions with elution volumes smaller than 406 mL and greater than 518 mL were not analyzed because they largely included peptides with high molecular weights, as well as free amino acids. ACE-I inhibitory activity (%) ranged from 22.66 to 45.96% (Figure 3) and from 36.91 to 55.83% (Figure 4) for 5–10 kDa and <1 kDa ultrafiltered fractions, respectively. The highest ACE-I inhibitory activity was observed in fractions *F*2 (45.96%) and *F*1 (55.83%) from 5–10 and <1 kDa *J. curcas* ultrafiltered fraction, respectively. Their molecular masses were approximately 0.9 kDa (indicative of 5 amino acid residues) and 1.8 kDa (indicative of 9 amino acid residues), respectively. The IC_50_ values for *F*2 (6.7 *μ*g/mL) and *F*1 (4.78 *μ*g/mL) were lower than those of *acetes chinensis* hydrolysates (Sephadex C-15 = 770–1590 *μ*g/mL) [30]. A similar behavior was observed to compare the results with Kim et al. [32] who separate by size exclusion chromatography peptide fractions below 3 kDa (IC_50_ = 500 *μ*g/mL) from textured and fermented vegetable protein (IC_50_ = 2190 *μ*g/mL). They purified peptide fraction with a molecular weight range of 500–999 kDa with IC_50_ values of 94 *μ*g/mL and that represented peptides of approximately 7 amino acids residues.

*J. curcas* protein hydrolysate has potential applications in the development of physiologically functional foods design to prevent and/or treat hypertension. An added benefit is the balanced amino acid profile of the peptide fractions, which makes them an appropriate protein source in human nutrition. It should be considered, however, that the *in vitro* ACE-I inhibitory potencies of peptides do not always correlate with their *in vivo* antihypertensive activities as quantified in SHR. This is because they must be absorbed and transported intact from the intestine to the blood stream (in the case of oral administration) and resist plasma peptidase degradation (in the case of oral and intravenous administration) to reach their target sites and exert an antihypertensive effect *in vivo*. Therefore, *in vivo* research is needed to determine to what extent any of the studied ACE-I inhibitory peptides may provide their antihypertensive activity *in vivo*.

Production of *J. curcas* protein isolates from defatted kernel meal is an alternative to reduce the contents of antinutritional and toxic components as well as a good substrate for the production of protein hydrolysates as a source of  bioactive peptides with beneficial biological activities. The immediate benefits of research on *J. curcas* protein hydrolysates and peptides would be in the area of nutrition and hypertension prevention and/or treatment. The information provided in this study may contribute to formulating strategies that could be used in practical situations, promoting human health, and making agriculture efficient, sustainable, and environmentally friendly.

## 4. Conclusions

Hydrolysis of *J. curcas* protein isolate with the Alcalase enzymatic system generated extensive hydrolysates with potential biological activity. This hydrolysis system produced low-molecular weight hydrolysates with ACE-I inhibitory activity and commercial potential as “health-enhancing ingredients” in the production of functional foods, contributing to enhance the financial viability and sustainability of a Jatropha-based biodiesel industry.

## Figures and Tables

**Table 1:** Amino acid contents in the <1 kDa and 5–10 kDa ultrafiltered fraction from *J. curcas* protein hydrolysates.

Amino acid	Composition (g/100 g)	
	*F* = 5–10 kDa	*F* < 1 kDa
Asx	6.66 ± 0.79^a^	12.83 ± 1.37^b^
Glx	7.37 ± 0.60^a^	13.92 ± 0.72^b^
Ser	6.27 ± 0.68^a^	5.03 ± 0.92^a^
His	4.86 ± 0.28^a^	3.17 ± 0.15^b^
Gly	6.52 ± 0.44^a^	3.40 ± 1.30^b^
Thr	4.30 ± 0.48^a^	3.60 ± 0.72^a^
Arg	11.83 ± 1.21^a^	7.86 ± 1.33^b^
Ala	7.60 ± 1.16^a^	4.88 ± 0.71^b^
Pro	8.32 ± 0.53^a^	4.85 ± 1.21^b^
Tyr	8.17 ± 1.48^a^	7.87 ± 1.50^a^
Val	1.20 ± 0.72^a^	1.58 ± 1.10^a^
Met	0.84 ± 0.21^a^	0.27 ± 0.17^b^
Cys	1.13 ± 0.06^a^	2.63 ± 0.39^b^
Ile	8.23 ± 1.47^a^	7.00 ± 1.32^a^
Trp	0.55 ± 0.24^a^	1.04 ± 0.02^b^
Leu	5.32 ± 1.41^a^	5.61 ± 1.33^a^
Phe	7.06 ± 1.15^a^	11.51 ± 1.22^b^
Lys	3.78 ± 0.69^a^	2.93 ± 0.44^a^

^a-b^Different superscript letters in the same row indicate statistical difference (*P* < 0.05).
